# New findings from the time trade-off for income approach to elicit willingness to pay for a quality adjusted life year

**DOI:** 10.1007/s10198-017-0883-9

**Published:** 2017-03-08

**Authors:** Arthur E. Attema, Marieke Krol, Job van Exel, Werner B. F. Brouwer

**Affiliations:** 1 iBMG, Erasmus University, P.O. Box 1738, 3000 DR Rotterdam, The Netherlands; 2 Merck B.V., Tupolevlaan 41-61, 1119 NW Schiphol-Rijk, The Netherlands

**Keywords:** Loss aversion, Time trade-off method, QALY, Utility of health and wealth, Willingness to pay, B41, D03, I10

## Abstract

In this paper we empirically investigate how to appropriately model utility of wealth and health. We use a recently proposed alternative approach to value willingness to pay (WTP) for health, making use of trade-offs between income and life years or quality of life, which we extend to allow for a more realistic multiplicative utility function over health and money. Moreover, we show how reference-dependency can be incorporated into this model and derive its predictions for WTP elicitation. We propose three experimental elicitation procedures and test these in a feasibility study, analysing the responses under different assumptions about the discount rate. Several interesting results are reported: first, the data are highly skewed, but if we trim the 5% lowest and highest values, we obtain plausible WTP estimates. Second, the results differ considerably between procedures, indicating that WTP estimates are sensitive to the assumed utility function. Third, respondents appear to be loss averse for both health and money, which is consistent with assumptions from prospect theory. Finally, our results also indicate that respondents are more willing to trade quality of life than life years.

## Introduction

Economic evaluations provide information on costs and effects of health technologies. Within economic evaluations, health effects are typically expressed in quality adjusted life years (QALYs). The QALY is a uniform outcome measure of health benefit that combines length of life with quality of life (QoL). By expressing health outcomes with a uniform measure, outcomes can be compared across different diseases and treatments, which can be helpful for decision makers in the process of making reimbursement decisions.

While operating under budgetary constraints and pressure, advisory bodies, such as the National Institute for Health and Clinical Excellence [1] in England and the National Health Care Institute [2] in the Netherlands, are searching for the shadow price of a QALY [3]. However, these two bodies use different shadow prices: NICE claims to base its shadow price upon forgone health [4], whereas the Dutch National Health Care Institute bases it upon the consumption value of health [2].

In the first case, the value of health is determined by comparing the expected health gains of a health intervention to the health that is likely forgone elsewhere due to the displacement of activities within a fixed budget (i.e. if a new therapy is reimbursed, the costs need to come from somewhere else within the health care budget). This approach is also labelled as adopting a health care perspective, focussing only (or primarily) on costs to the health care sector and the health effects of an intervention. Cost-effectiveness analyses may suffice to prioritize healthcare in this case, operating under an exogenous budget constraint that is imposed by a higher authority [4, 5]. In general, the decision rule then indicates that only when the health gained exceeds the health displaced (abstracting from possible equity considerations), a new intervention should be adopted. Within this framework, it is not possible to judge whether the budget itself has been set appropriately.

In the second case, the value of health is determined by assessing the amount of consumption that individuals are willing to give up to improve health [4]. This approach relates to adopting a societal perspective in performing economic evaluations, taking into account the broader societal costs and benefits of health interventions. Countries considering using this decision framework require a monetary estimate of the (consumption) value of health. The decision rule then becomes that the monetary value of the health produced (welfare gained) should exceed the monetary value of the costs (welfare sacrificed). As long as this rule is followed in adopting and applying technologies, the appropriate budget follows from these decisions. In this paper we focus on the estimation of the consumption value of health and, hence, we seek to estimate the monetary value of a QALY.

Two kinds of willingness to pay (WTP) approaches have frequently been used to estimate the monetary value of a QALY. The first approach has been to elicit the WTP for a reduction in the risk of death and then calculate the value of a life, from which the monetary value of a QALY can be inferred [6–9]. The second approach has been to elicit the WTP for changes in health status directly [10–21].

Few WTP studies have investigated the role of reference-dependency, which has often been demonstrated to play a considerable role in people’s decisions and valuations [22–25]. Reference-dependency is part of prospect theory and implies that individuals consider a reference point and frame outcomes as gains and losses relative to this reference point [26]. Furthermore, losses are often given more weight than gains of similar size, a phenomenon which has been termed loss aversion [27]. If reference-dependency is not taken into account, the valuation of health obtained from a WTP study may be affected considerably by the particular framing used and is potentially distorted by loss aversion. For example, in a recent paper, Holte et al. [28] tested reference-dependency in WTP among physicians using both a contingent valuation and a Discrete Choice Experiment (DCE), and found that they value losses from their current income 3 times higher than equivalent gains.

Tilling et al. [29] suggested an alternative approach to estimate the monetary value of a QALY, based upon a time trade-off (TTO) exercise. In this method, people are asked to choose between living longer (in some fixed health state) with less income and living shorter (in that same health state) with more income. Thus, a trade-off is made between length of life (in a particular health state) and income, which allows investigation of the implicit monetary value given to QALYs. Tilling et al. [29] estimated WTP assuming an additive lifetime utility function, which may be too restrictive [30–35]. Therefore, in this paper we investigate empirically how to model health and wealth more appropriately. To this end, we assembled data in a representative sample of the Dutch population, using a multiplicative utility function in the computation of WTP and allowing for reference-dependence and loss aversion. In addition, besides longevity we also include a variation in QoL in order to explore whether these different response scales generate the same willingness to pay for a QALY. Finally, we compare performance of different specifications of the utility function, with and without discounting.

In what follows, we present our model and extend it to include reference-dependency. “Experiment” gives details of the experiment and “Results” present its results. Finally, “Discussion” ends the paper with a discussion of the results.

## Methods

In the previous ‘TTO for income-study’ [29], an additive function *W*(.) over healthy life years (*H*) and income (*Y*) was assumed:

1 $$W\left( {H,\;Y} \right) = U\left( H \right) + Y.$$

That is, individuals derive value from their lifetime and have a linear utility function over income. This specification was used earlier by Eeckhoudt et al. [36].^1^ The advantage of this function is that it becomes straightforward to elicit a monetary value from the utility of perfect health. The pitfall is that it is descriptively less accurate. In particular, assuming this utility function implies independence of consumption utility from the level of health, which was one of the ‘impossibility theorem criteria’ set out by Dolan and Edlin [38]. Moreover, the empirical literature tends to reject this assumption in favour of a multiplicative utility function over health and income. Indeed, there is evidence that marginal utility of wealth increases with health and longevity, which is impossible under an additive function [30–33, 35].^2^ We therefore study the following utility function over health and income:

2 $$W\left( {H,Y} \right) = D\left( t \right) \times Q \times V(Y),$$

with $D\left( t \right) = \mathop \sum \nolimits_{t = 1}^{T} \delta (t)$ representing the sum of the discount factors $\delta (t)$ for each period *t* until the final period under consideration *T*,^3^ and *Q* the QoL experienced during all periods between *t* = 1 and *t* = *T* (i.e. a chronic health state). We take *Q* to be on the usual cardinal scale with 0 indicating a health state as bad as being dead and 1 indicating full health. Bleichrodt and Quiggin [40] have given the axiomatic foundations for this function. The simplest configuration would be to take both *D*(*t*) and *V*(*Y*) to be linear (i.e. $\delta (t)$ = 1 for all *t* and *V*(*Y*) = *Y*), but this lacks realism. It is more likely that marginal utility decreases with income, i.e. *V*ˈ(*Y*) < 0. Here, we model this by considering a power utility function $V\left( Y \right) = Y^{\alpha }$, with α as a measure of the utility curvature of income and $V\left( Y \right) = \ln (Y)$ for *α* = 0 [41]. Decreasing (constant, increasing) marginal utility of income is reflected in this function by *α* < 1 [=1, >1]. Therefore, our lifetime utility function will take the form:

3 $$W\left( {H,Y} \right) = D(t) \times Q \times Y^{\alpha } .$$

Empirical support for this function was provided by Levy and Nir [42], who used a special case of this function where *V*(*Y*) = ln (*aY*) (i.e. *α* = 0 in Eq. 3 and a scaling parameter a). In the following sections, we present the predictions stemming from the multiplicative model. In addition, the predictions according to the additive model are given in “Appendix A”.

### Income levels

Before the experiment started, subjects were, among other things, asked for: their current net household income (called *C* hereafter), the net income that would be sufficient to just make ends meet while staying in their current house (subsistence income, called* S* hereafter), and the net income they would need to be able to live a comfortable life without any worries (luxury income, called* L* hereafter).^4^

### The TTO for income approach

In TTO1, respondents were asked to choose to live *T* = 10 more years in their current health state *Q* (as measured by a visual analogue scale at the beginning of the experiment) and their current salary *C* or to live an amount *X*_1_ ≤ 10 years in their current health state *Q* but with their higher luxury income *L*.

Suppose you can choose between the following two options:

Option A

“You live for 10 years in your current health state with a net monthly income of [*C*/12], without any changes to it. Then you die.”

Option B

“You live for *X* yearsin your current health state with a net monthly income of [*L*/12], without any changes to it. Then you die.”

### TTO1: Trading life years to achieve an income gain in current health

Hence, TTO1 elicited the number of life years *X*_1_ such that the subject would be indifferent between (10 years, *C*) and (*X*_1_ years, *L*). Under the multiplicative model (Eq. 3), this would result in the following equality:

4 $$D(10) \times Q \times C^{\alpha } = D(X_{1} ) \times Q \times L^{\alpha } .$$

From this, we can compute an estimate of *α*:

5 $$\alpha = \frac{{\ln \left( {D(10)} \right) - \ln (D(X_{1} ))}}{\ln \left( L \right) - \ln (C)}$$

with *α* > 0. Having this estimate, we can continue to infer an estimate of the WTP for 1 year in full health [WTP(YFH)]. For example, we can estimate the income *Y* such that, given the estimate of α from Eq. 5, living 9 years with this income would give equal (remaining) lifetime utility as the initial scenario with 10 years and income C:

6 $$D(10) \times C^{\alpha } = D(9) \times Y^{\alpha } \Leftrightarrow Y = \left( {\frac{D(10)}{D(9)}} \right)^{1/\alpha } \times C.$$

WTP for a healthy life year is then given by the additional lifetime income people demand in return for reducing life by 1 year, corrected for their QoL:

7 $${\text{WTP}}\left( {\text{YFH}} \right) = \frac{{\left( {D(9)Y - D(10)C} \right)}}{Q} = \frac{{\left( {D(9)\left( {\frac{D(10)}{D(9)}} \right)^{1/\alpha } - D(10)} \right)C}}{Q}.$$

Equation A2 in Appendix A gives the expression for WTP under the additive model with *X* = *X*_1_.

In TTO2, respondents were asked to choose to live *T* = 10 more years in their current health state *Q* and with a lower salary *S*, or to live an amount *X*_2_ ≤ 10 years in their current health state *Q* but with current income* C*.

Suppose you can choose between the following two options:

Option A

“You live for 10 years in your current health state with a net monthly income of [*S*/12], without any changes to it. Then you die.”

Option B

“You live for *X* years in your current health state with a net monthly income of [*C*/12], without any changes to it. Then you die.”

### TTO2: Trading life years to achieve an income gain in current health

TTO2 gives the estimates of α and WTP for the multiplicative [additive] model as provided in Eqs. 5 and 7, (A2), with *C* replaced by *S*, *L* replaced by *C*, and *X*_1_ by *X*_2_.

A third possibility of eliciting the monetary value of a QALY is a new variation to the common TTO for income procedure: the quality trade-off (QTO). This procedure varies QoL instead of life duration. Suppose we apply QTO with *T* = 10 years in full health and income *C*, and we ask for the QoL score *X*_3_ with *T* = 10 years with income *L* rendering indifference. Health status is described on a 10-point scale, with 10 representing perfect health and 0 a health state as bad as being dead. This is comparable to a visual analogue scale (VAS), which is frequently used for health status measurements. Parkin and Devlin [43] give advantages of using the VAS in cost-utility analyses.

### QTO: Trading quality to achieve an income gain during 10 remaining years

Suppose you can choose between the following two options:

Option A

“You live for 10 years in a perfect health state (10 on a scale of 0–10) with a net monthly income of [*C*/12], without any changes to it. Then you die.”

Option B

“You live for 10 years in moderate health (*X* on a scale of 0–10) with a net monthly income of [*L*/12], without any changes to it. Then you die.”

For the sake of convenience, but without affecting the results, we transformed *X*_3_ to a 0–1 scale by dividing the answer by 10. Hence, *X*_3_ has a range of 0 (death) to 1 (perfect health). If there is no reference-dependency, this indifference can again be evaluated by Eq. 3, yielding:

8 $$D(10) \times 1 \times C^{\alpha } = D(10) \times X_{3} \times L^{\alpha } ;$$

9 $$\alpha = \frac{{ - \ln \left( {X_{3} } \right)}}{\ln \left( L \right) - \ln (C)}.$$

Because according to the QALY model *T* and *Q* are fully exchangeable, meaning that living 10 years with QoL 9 is equivalent to living 9 years with QoL 10, solving Eq. 8 for WTP(YFH) yields the same result as Eq. 7. Therefore, this model predicts WTP and *α* to be the same in TTO1 and QTO. In other words *X*_1_ is predicted to be equal to *X*_3_.

### Accounting for reference-dependent preferences

A large body of evidence has emerged suggesting that people deviate from several rationality assumptions underlying neoclassical economic theory. One such deviation is that individuals tend to behave according to prospect theory [25, 26, 44]. In particular, they often form reference points and handle gains and losses as seen from this reference point differently. There is evidence that this behaviour also occurs in health-related decision making [45–47]. In order to accommodate this possibility, we analysed our data under this assumption from prospect theory as well.

Preferences become reference-dependent if we assume prospect theory, which requires separate formulations for gains and losses. In particular, we investigated reference-dependency by the model proposed by Shalev [48], which for income culminates into:

10 $$U\left( Y \right) = \left\{ {\begin{array}{*{20}c} {u(Y)} \\ {u(Y_{0} + \lambda_{M} \left( {Y - Y_{0} } \right))} \\ \end{array} } \right.\quad \begin{array}{*{20}c} {{\text{if }} Y \ge Y_{0} } \\ {{\text{if }} Y < Y_{0} } \\ \end{array} ,$$

with $\lambda_{\text{M}}$ a loss aversion index for monetary outcomes and Y_0_ the status quo. Although the utility function may be different for gains and losses, e.g. *u*(*Y*) = *Y* ^*α*^ for gains and *u*(*Y*) = −(−*Y*)^*β*^ for losses, with *α*, *β* > 0, for simplicity we assume they are the same. Extending this model to health yields:

11 $$U\left( H \right) = \left\{ {\begin{array}{*{20}c} {u(H)} \\ {u(H_{0} + \lambda_{H} \left( {H - H_{0} } \right))} \\ \end{array} } \right.\quad \begin{array}{*{20}c} {{\text{if }} H \ge H_{0} } \\ {{\text{if }} H < H_{0} } \\ \end{array} ,$$

with $\lambda_{H}$ a loss aversion index for health outcomes.

In the last part of this section we describe the three experimental procedures that will be applied in this study and two hypotheses to be tested based on these procedures. First, suppose we apply TTO1 again with the same stimuli. According to prospect theory, respondents then have to trade off a gain in income against a loss in lifetime. If we assume {10 years, *C*} to be the reference point, this involves comparing the status quo against a mixed prospect, which would be evaluated by:

12 $$D(10) \times Q \times C^{\alpha } = \left[ {D(10) + \lambda_{H} \left( {D(X_{1} ) - D(10)} \right)} \right] \times Q \times L^{\alpha } .$$

Solving this expression for *X*_1_ gives:

13 $$X_{1} = D^{ - 1} \left[ {\frac{D(10)}{{\lambda_{H} }}\left[ {\left( {\frac{C}{L}} \right)^{\alpha } + \lambda_{H} - 1} \right]} \right].$$

which is increasing in $\lambda_{H}$. Therefore, *X*_1_ will be higher for people who are loss averse ($\lambda_{H}$ >1) than for people who are loss neutral ($\lambda_{H}$ = 1). In the classical approach described in “Methods” loss aversion is ignored, implicitly assuming $\lambda_{H}$ = 1. Consequently, the effect of loss aversion will be picked up by our estimate of α (Eq. 5), which is decreasing in *X*_1_ and, hence, will be lower if people are loss averse than if they are not. As derived in “Appendix B”, the real estimate of α is given by:

14 $$\alpha = \frac{{\ln \left( {D\left( {10} \right)} \right) - \ln \left( {D\left( {10} \right) + \lambda_{H} \left( {D\left( {X_{1} } \right) - D\left( {10} \right)} \right)} \right)}}{\ln \left( L \right) - \ln (C)},$$

which requires knowledge of $\lambda_{H}$. Therefore, because our estimated α is decreasing in *X*_1_, and *X*_1_ increases with $\lambda_{H}$, the classical approach can be expected to generate an underestimation of the true α in case of loss aversion and, hence, an overestimation of WTP for a QALY (Eq. 7).

Now let us reconsider TTO2 in case of prospect theory. If we assume {10 Years, *C*} is still the reference point, the first option now entails a loss in income, whereas the second option still entails a loss in health. In other words, we are now comparing a loss in the monetary domain to a loss in the health domain. Indifference between the two options can then be evaluated by:

15 $$D(10) \times Q \times \left[ {C + \lambda_{M} (S - C)} \right]^{\alpha } = \left[ {D(10) + \lambda_{H} \left( {D(X_{2} ) - D(10)} \right)} \right] \times Q \times C^{\alpha } ,$$

which gives a different solution for *X*_2_ than we had for *X*_1_ in the first procedure (Eq. 13):

16 $$X_{2} = D^{ - 1} \left[ {\frac{D(10)}{{\lambda_{H} }}\left[ {\left( {\frac{{C + \lambda_{M} (S - C)}}{C}} \right)^{\alpha } + \lambda_{H} - 1} \right]} \right],$$

*X*_2_ is increasing in $\lambda_{H}$ again, but at the same time decreasing in $\lambda_{M}$. In other words, the two loss aversion coefficients are opposing forces in determining *X*_2_ and the qualitative effect of loss aversion on *X*_2_ will therefore depend on the relative values of $\lambda_{H}$ and $\lambda_{M}$. Consequently, the estimate of α (Eq. 5) is expected to be higher in TTO2 than in TTO1. Since Eq. 5 provides an underestimation of α in TTO1, the amount of the underestimation would be reduced in TTO2, and may even change into an overestimation if $\lambda_{H}$ is high enough.

### Hypothesis testing

We therefore formulate the following hypothesis:

#### Hypothesis 1

The estimated power coefficient of the utility function will be lower in TTO1 than in TTO2 (TTO1 vs TTO2): *α*_1_ < *α*_2_

The hypothesis will be tested within-subjects using a paired *t*-test (for means) and a Wilcoxon signed ranks test (for medians) on *α*_1_ and *α*_2_. A confirmation of this hypothesis would be a violation of the multiplicative function as formulated in Eq. 3 and could be explained by prospect theory or another parametric shape of the utility functions, as further explained in the “Discussion” section. A rejection of Hypothesis 1 would imply that the classical theory cannot be falsified.

If prospect theory holds, respondents have to trade off a gain in income against a loss in QoL. Assuming {10 years in full health, *C*} to be the reference point, this again involves comparing the status quo against a mixed prospect, which under the multiplicative model would be represented by:

17 $$D(10) \times 1 \times C^{\alpha } = D(10) \times 1 \times \left[ {1 + \lambda_{Q} (X_{3} - 1)} \right] \times L^{\alpha } .$$

This expression can be solved for *X*_3_:

18 $$X_{3} = \frac{1}{{\lambda_{\text{Q}} }}\left[ {\left( {\frac{C}{L}} \right)^{\alpha } + \lambda_{\text{Q}} - 1} \right].$$

Comparing Eqs. 13 to  18, it becomes evident that *X*_1_ and *X*_3_ are expected to differ only to the extent that loss aversion for QoL differs from loss aversion for life duration, and to the extent that people discount the future.

It is important to obtain information about the amount of loss aversion in both life duration and QoL, since many preference elicitation tasks, such as TTO, standard gamble or WTP involve the reduction of one or both of these outcomes. There is very limited evidence on the amount of loss aversion for life duration and QoL, though [45, 49]. Consequently, based on the current literature, we cannot make a confident prediction as to whether loss aversion is stronger for life duration or for QoL. Intuitively, people may be more reluctant to give up lifetime, which would translate into more loss aversion for life duration than for QoL, but no firm evidence is available on this point. Consequently, our second hypothesis is the following:

#### Hypothesis 2

There is no difference in the loss aversion coefficient for life duration and quality of life (TTO1 vs QTO): $\lambda_{H}$ = $\lambda_{Q} .$

A confirmation of this hypothesis implies that agents are equally loss averse for these two outcomes, whereas a rejection would suggest they are not. We will test this hypothesis by comparing *X*_1_ and *X*_3_ using a paired *t*-test and a Wilcoxon signed ranks test. *X*_1_ and *X*_3_ are predicted to be equal if $\lambda_{H}$ = $\lambda_{Q} ,$ as derived earlier.

## Experiment

### Subjects

A total of 550 subjects, representative for the Dutch adult population in terms of gender, age and level of education, participated in the experiment. The study presented here was part of a larger experiment conducted in 2013 that included data collection for two other (unrelated, yet unpublished) studies investigating positional concerns in health.

### Procedure

The procedure to arrive at an estimate of *X* consisted of (a maximum of) three steps. In the first choice between options A and B, *X* was always equal to 10 years (life duration part) or 10 QoL points (QoL part). Because monotonicity implies dominance of option B in this situation, we would expect respondents to opt for B here. In case one chose A, we asked whether they really preferred 10 years with lower income *C* to 10 years with higher income *L*. If so, these respondents were viewed as people who “are not willing to play the game” and a missing value was saved for *X*. Otherwise, they received the original question anew. If respondents were indifferent, a value of 10 was saved for X. If B was chosen, *X* was randomly lowered to 3, 5, or 7 years/QoL points. The respondent could then choose A or B again or express indifference. In case of indifference, the provided value of *X* was the elicited indifference point. If A or B was chosen, the respondent had to indicate the value of *X* such that A and B were equally attractive to them by using a scroll bar, where the range of the scroll bar was censored by the previous choice. For example, if the respondent received *X* = 3 in the second choice and then opted for A, the scroll bar was censored between 3 and 10, whereas it was between 0 and 3 if they opted for B. The order of the WTP questions was the same for all respondents: TTO1 was elicited first, followed by TTO2 and QTO.

The experiment was conducted by a professional internet sampling company (Survey Sampling International). This company has much experience with internet surveys and a large representative database of subjects. The subjects were rewarded with a small monetary amount to be given to a charity fund of their choice, upon completion of the questionnaire.

Current, subsistence and luxury income were measured on a categorical scale (with “999 € or less” as the lowest category, “8000 € or more” as the highest category, and eleven 500-€ intervals in between). We used the midpoint of the chosen scale as the amount (i.e. *C*, *S* or *L*) to be used in TTO questions. Whenever someone expressed subsistence income to be above current income (38.9%), or luxury income below current income (10%), we replaced these values in the TTO questions in order to enable sensible trade-offs. In particular, *S* was replaced by half of current income and *L* was replaced by twice the amount of current income.

It was possible not to trade off any life years or quality of life. In the remainder of this paper, a respondent who behaves in this fashion in a task is termed a non-trader in that particular question. Furthermore, it was also possible to trade so many years/quality of life that the resulting WTP was negative. If such a result occurs, we speak of *over*-*trading* in the particular task.

### Analysis

As pointed out by Gyrd-Hansen and Kjær [50], there tends to be a lot of heterogeneity in WTP for QALY estimates. They demonstrate that, because of this heterogeneity, the choice of the analytical approach can make for a large difference in WTP estimates. They compared the aggregated or ‘ratio of means’ approach (i.e. sum of the individual WTP estimates divided by the sum of the considered QALY gains) with the disaggregated or ‘mean of ratios’ approach (i.e. the mean of ratios of the WTP and the associated QALY gain for each individual separately), and observed large differences in the results. One of the reasons was that in the disaggregated approach it was not possible to include non-traders, because their QALY gain was zero. Our data contains a lot of non-traders: 247 (44.9%), 180 (32.7%) and 148 (26.9%) for TTO1, TTO2 and QTO, respectively (see Table 1). Indeed, a disadvantage of the current method is that, if we would use the disaggregated approach, we do not obtain information about the monetary value of health for a significant fraction of the respondents. In this study we therefore use the aggregated approach. This approach allows the inclusion of the valuations of the non-traders. However, the results from the disaggregated approach are shown in “Appendix C”.

**Table 1:** Overview of WTP classifications

		WTP1 (L-C)	WTP2 (C-S)	WTP3 (L-C QoL)
A	Non-traders	247 (44.9%)	180 (32.7%)	148 (26.9%)
B	Over-traders; negative WTP	77 (14%)	111 (20.2%)	0 (add); 151 (27.5%)
C	Over-traders; trading off all years/quality	1 (0.2%)	8 (1.5%)	2 (0.4%)
D	Zero WTP	12 (2.2%)	59 (10.7%)	0
E	*S* = 0^a^	0	2 (0.4%)	0
F	Net sample size aggregated approach	550	550	550

^a^Excluded from analysis

The analyses were performed assuming two different scenarios: zero discounting and a conventional 3% annual discount rate. The main findings were not sensitive to the choice of the discount rate. Both analyses are reported below.

Our design allowed for a crude test of sensitivity to scope, both at the inter-respondent and the intra-respondent level. Regarding the former, we could test whether respondents with a higher difference between* C* and* L* also gave up more life years and quality of life. This was performed by Kendall’s *τ* test on the correlation between* L*–*C* [*C*–*S*] and 10 − *X*_1_ and 10 − *X*_3_ [10 − *X*_2_] (i.e. the number of years/QoL points traded). At the intra-respondent level, any difference between the increase from *S* to *C* and the increase from *C* to *L* could similarly be used to test for sensitivity to scope. We accomplished this by computing the ratio of (*L*–*C*) − (*C*–*S*) = *L* + *S*-2*C* to (*X*_1_–*X*_2_). This ratio should be positive if respondents are sensitive to scope. However, it should be noted that, contrary to an ideal test of sensitivity to scope, the starting levels are different. Hence, it may be that respondents are sensitive to the amount of income to be gained, but still do not trade more life years for a higher income gain if the starting level is much higher (i.e.* C* vs* S*), because of diminishing marginal utility of income.

Our dataset enabled a straightforward, although admittedly restricted, performance test of different utility functions. This was accomplished by computing the squares of the individual differences between WTP1 and WTP2 for several model specifications (i.e. the additive model and the multiplicative model both with power and exponential utility for income, with and without reference-dependence, and with and without 3% discounting) and testing for differences in this squared error between models (Wilcoxon signed ranks tests).^5^

## Results

Tables 7 and 8 in Appendix D present some demographic variables of our sample, as well as descriptive statistics of *X*_1_, *X*_2_ and *X*_3_. The numbers indicate representativeness for the Dutch adult population according to age, gender and education.

**Fig. 1:**
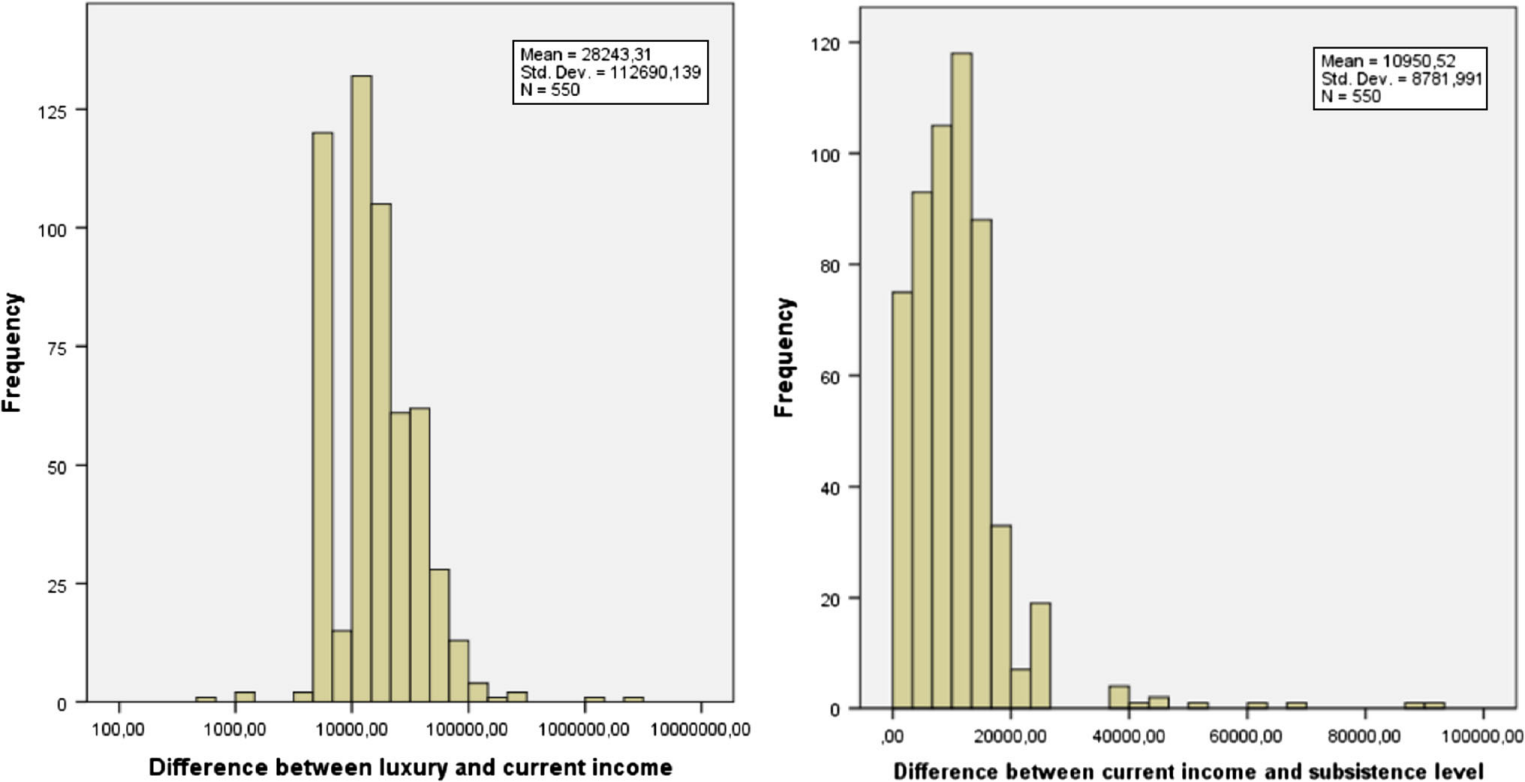
Distributions of income changes received by the respondents

The mean current net household income (*C*/12) of the respondents was 2152.27 € per month (range 500 €–8500 €, SD 1310.29 €). Furthermore, their reported mean monthly subsistence income level (S/12) was 2080.42 € (range 0 €–20,000 €; SD 1204.67 €), and the mean monthly luxury income level (*L*/12) was 3706.51 € (range 0 €–203,039 €; SD 9685.95 €). The percentage of respondents stating *S* ≤ *C* was 61.1%, whereas 90.0% reported *L* ≥ *C*. Figure 1 shows the distributions of the income improvements respondents faced in the experiment. This shows there is a lot of heterogeneity.

Table 1 classifies the respondents in different groups: non-traders, over-traders (i.e. causing a negative WTP) and respondents with zero WTP; a high number of non-traders appeared in all three tasks.

Table 2 gives the estimates obtained under the multiplicative model. This table shows a similar pattern across methods. The outliers are less influential in the aggregated approach than in the disaggregated approach (see Appendix C, Table 6), giving much more conservative estimates. In order to remove the inflating effect of the outliers, we also analysed the data using a trimmed dataset, where we removed the 5% highest and 5% lowest WTP ratios.

**Table 2:** WTP estimates (in €, 2013) multiplicative model (aggregated approach)

	WTP1 (L–C)				WTP2 (C–S)				WTP3 (L–C QoL)	
	*α*_1_ no discounting	*α*_1_ 3% discounting	WTP1 no discounting	WTP1 3% discounting	*α*_2_ no discounting	*α*_2_ 3% discounting	WTP2 no discounting	WTP 2 3% discounting	*α*_3_	WTP3
Mean	0.30	0.26	95,221	85,405	0.63	0.55	12,616	12.783	0.46	33,842
Median	0.08	0.07	580,061	508,705	0.81	0.71	4299	5438	0.55	18,648
Mean (trimmed data)^a^	0.33	0.29	75,286	68,258	0.57	0.50	14,808	14,537	0.55	22,340

^a^5% upper and lower value

The formal tests of our hypotheses give the following results.

Hypothesis 1. We observe (see Table 2) *α*_1_ to be lower than *α*_2_ (*p* < 0.01), which is consistent with our prediction resulting from loss aversion. Related to this finding, the median WTP is higher for TTO1 than for TTO2 (*p* < 0.01), indicating individuals are willing to give up more lifetime to move from a subsistence income to their current reference level income, than to move from their current reference level income to a luxury income.

Hypothesis 2*. X*_1_ is higher than *X*_3_ (Table D2, *p* < 0.01), indicating that loss aversion is stronger for life duration than for QoL.

### Sub-group analyses

We also performed several sub-group analyses. In particular, we tested whether there were differences in the proportions of non-traders and over-traders according to gender, age, and educational background. There were no differences between men and women (*χ* ^2^ test, *p* > 0.67). For education, a *χ* ^2^ test showed no effect of education for TTO1 (*p* = 0.17) and QTO (*p* = 0.055), but it did for TTO2 (*p* < 0.01). Specifically, more highly educated people had a greater tendency to be non-traders and a lower tendency to be over-traders. Correlations between age and WTP were not significant for TTO1 and TTO2 (Kendall’s τ test, *p* > 0.25), but there was a significant negative correlation between age and WTP for QTO (Kendall’s *τ* test, *p* < 0.05). Finally, we tested the effects of the above socio-demographic characteristics simultaneously by conducting logistic regressions on the various WTP measures. The results are presented in Table 3 and indicate a similar pattern.

**Table 3:** Logistic regressions

	TTO1				TTO2				QTO			
	WTP1 negative		WTP1 infinite		WTP2 negative		WTP2 infinite		WTP3 negative		WTP3 infinite	
	Coefficient	SE	Coefficient	SE	Coefficient	SE	Coefficient	SE	Coefficient	SE	Coefficient	SE
Constant	−1.39*	0.61	−1.17**	0.44	−1.53**	0.54	−1.57**	0.47	−0.45	0.48	−2.58**	0.51
Age	0.00	0.01	0.01	0.01	0.004	0.01	0.004	0.01	0.001	0.007	0.02**	0.01
Female	−0.11	0.25	0.21	0.17	0.05	0.21	0.14	0.18	−0.11	0.19	0.17	0.20
Medium education	−0.25	0.29	0.41	0.22	−0.08	0.10	0.51*	0.24	−0.47*	0.23	0.58*	0.25
High education	−0.54	0.33	0.61**	0.23	−0.26	0.29	0.99**	0.25	−0.62*	0.25	0.74**	0.27

* Significant at the 5%-level

** Significant at the 1%-level

### Sensitivity to scope

Our results on sensitivity to scope are mixed. Between-respondents, we observe a positive correlation between *L*–*C* and the number of years traded (10-*X*_1_) (Kendall’s *τ* test; *p* < 0.05), but no significant correlations for the other question (*X*_2_, *p* = 0.72). Within-respondents, 167 [179, 170] have a positive [negative, zero] ratio of *L* + *S*-2*C* to (*X*_1_-*X*_2_).^6^ Therefore, there is not much evidence for sensitivity to scope within-subjects. However, as noted before, this is no evidence of absence of the scope effect per se, because of the varying starting levels. We should also bear in mind that these results partly follow from the main test results: if there were perfect scope effects, WTP would be the same for all questions, and we observed in the previous part of this section that it is not.

### Performance test

The multiplicative model with power utility for income, loss aversion, and a 3% discount rate had a lower squared error than the other specifications (*p* < 0.01 for all comparisons). Of course, a more accurate test would have loss aversion coefficients and discount functions elicited at the individual level, but still our results suggest that models with state- and reference- dependent models perform better than additive and non-reference-dependent models, and that discounting of future outcomes should be taken into account in WTP estimations.

## Discussion

This research set out to explore a novel method of valuing life years by means of trading life years for income. We applied three different procedures to elicit WTP with this method, under different assumptions about the utility functions for health and money. Moreover, we extended the model to incorporate reference-dependency and derived its prediction for each of these procedures.

Our trimmed WTP estimates give numbers that are comparable to estimates found in the literature [6, 13, 21, 51], although the high variation across procedures indicates a high susceptibility to the particular procedure employed. Likewise, the differences between models show the large influence of the particular assumptions about the utility functions for life duration and consumption on WTP estimates. Regarding the former, we find a difference in WTP between two procedures in the direction predicted by prospect theory. This result is confirmed by a test showing that a multiplicative, reference-dependent model with discounting has the highest predictive power. Furthermore, we observe less non-trading when using QoL instead of life duration as response scale, although this does not necessarily translate into higher WTP for a healthy life year.

One of the limitations of this study was the high number of non-traders. Non-willingness to trade may be a sincere preference or an expression of protest against the nature of the exercise, but part of it may also be the result of the magnitude of our trade unit. The minimum amount to be traded was 0.1 years, which is approximately 5 weeks. So, if people were only prepared to sacrifice, say, 2 weeks, 0 was closer to this amount than 0.1 years. These respondents would then appear to have an infinite WTP, whereas in reality their WTP is finite (albeit high).

Although many respondents did not trade at all, only about 25% of these non-traders expressed indifference between 10 years with the lower income and 10 years with the higher income, which would be the implication of non-trading. The other 75% preferred 10 years with the higher income, but picked the highest possible answer in the slider (i.e. 10 years with the higher income). Hence, it seems that these respondents had some other reason to refuse any trading than being indifferent between earning a lower or a higher income. Explanations may be that they attempted to ‘improve their position’ or because their indifference value was between 9.9 and 10 years, which could not be expressed in our questionnaire (see above). Future research may therefore experiment with other designs such as only presenting binary choices or not applying sliders. Moreover, such research may more directly address the motivation of respondents for certain response patterns. Finally, more personalized information could be given to respondents, accompanied by a feedback module, in order to reduce misunderstandings of the choice task.

The difference in non-trading behaviour between TTO1 and TTO2 may also have been caused by the size of the difference between current and luxury income, versus the difference between subsistence and current income, which of course differed between subjects. The former difference was higher on average than the latter. Consequently, respondents were more likely to give up lifetime in the current-luxury trade-off than in the subsistence-current trade-off.

A second limitation was that a substantial part of the respondents traded too many life years, leading to a negative WTP for a life year. This finding may be caused by respondents not seriously engaging in the task, or difficulties with comprehending the task (despite our explanation of the fact that their answer implies their total income will be lower and their life span shorter), the latter being underlined by a lower proportion of over-trading among higher educated respondents. However, it may also be the result of a true preference for a high income per period. Obviously, a negative WTP is nonsensical, as it implies these people would not want to live an additional year in full health, even if it would cost them no money at all. However, a possible rationalization for this behaviour might be that individuals derive such a high amount of utility from having a high(er) income *per month* that they prefer a short life with a high monthly income over a longer life with more *total income* but a lower *monthly income*. This argument would translate into a composite utility function that incorporates utility of income instead of utility of wealth. Finally, the over-trading may be caused by a high amount of discounting. In our analysis we only considered discount rates of 0 and 3%, but if in reality respondents give less weight to their future life years,^7^ this may have erroneously caused a negative WTP in our study. This underlines the necessity to elicit discounting future health alongside a measurement of WTP in future work. Hence, more research is required to sort out these questions.

Third, our results reveal that respondents tend to pick the highest amount of the range in the scroll bar question, resulting in a multi-peaked answer distribution. This observation points toward some kind of preference construction, where respondents are influenced by the initial question. That is, they may be subject to an anchoring bias, as reported earlier in TTO and WTP studies [25, 52, 53]. Furthermore, their indifference value may not necessarily represent a true indifference, but instead a wish of subjects to improve their position [54]. Such an erroneous perception of the task as a bargaining task would imply an underestimation of the amount of lifetime respondents are willing to trade off, and, hence, and overestimation of WTP. Because this kind of behaviour could be foreseeable according to previous research on TTO [55], we implemented three different stimuli in the second choice of each task (i.e. 3, 5 and 7 years/QoL points, cf. “Experiment”).

Fourth, the TTO2 and QTO versions generated significantly fewer respondents who were indifferent between 10 years with income *L* [*C*] and 10 years with income *C* [*S*], or who even preferred the latter to the former option, than the TTO1 version. Given that TTO2 and QTO were always asked after TTO1, this finding could be due to a learning effect. Future research randomizing the order of these tasks is needed to test this possibility.

Another criticism may be that the changes in income that respondents faced could be view as non-marginal, which is not fully in accordance with the theoretical underpinnings of WTP for a QALY or the value of a statistical life. However, using smaller income changes would have evoked even more non-trading than already found in this study. Moreover, there was a lot of heterogeneity around the income increases. The difficult trade-off between non-trading and non-marginal changes in the stimuli could be considered a weakness of the studied method. Future studies are called for to test the robustness of this method to smaller income changes. Such a study should arguably also use a more refined response scale, e.g. expressed in days, hours or even minutes, in order to be sensitive to such small income changes. We also advocate future research to perform a head-to-head comparison of the presented method with the classical WTP method to directly compare their estimates.

Like in common TTO exercises, our method comprises of a trade-off between two certain options. The traditional TTO method involves a number of assumptions and limitations [56–58]. The key assumptions are constant proportional trade-offs, risk neutrality with respect to life years, and mutual utility independence [59]. Our findings indicate that QoL and income are closer substitutes than longevity and income. This may be caused by people being reluctant to give up longevity in general, especially when life expectation is not very long. For example, several TTO studies have found respondents to violate constant proportional trade-offs because they were willing to give up relatively more life years for longer life expectancies than for shorter ones [60–62]. In addition, Pinto-Prades et al. [63] reported that people gave more weight to QoL than to longevity in valuing end-of-life QALYs. These findings raise serious questions, since they imply, for instance, that the standard TTO method is not valid. More research is required to investigate this violation in more detail.

Finally, although we allowed for discounting in our analysis, we had to assume all subjects discounted at the same, constant rate. Ideally, in order to capture heterogeneity in discounting behaviour, future research should separately measure discounting at the individual level, perhaps also allowing for the possibility of non-constant discounting. However, this comes at the expense of higher response burden and we suspect it will not affect the within-subject WTP comparisons.

Notwithstanding these shortcomings, several conclusions and areas for future research emerge from our experiment. First, WTP is sensitive to both the amount of the income compared and to the currency used to trade off health for money (i.e. life years or QoL). Second, large differences in WTP result from making different assumptions regarding the lifetime utility function, stressing the need to obtain a valid measurement of the parametric shape of this function. Third, the high numbers of infinite and negative WTP estimates indicate that the procedure used in this study has drawbacks (like common WTP approaches). The presence of non-traders is inherent to the WTP and TTO approaches in general and hard to resolve. The presence of over-traders is specific to the current method.

Our findings were consistent with Hypothesis 1 (i.e. *α*_1_ < *α*_2_), but loss aversion need not be the only reason for this. One other possibility would be that the multiplicative model is valid but that it needs to be accompanied by a nonlinear utility function over life years [64–67]. For instance, if individuals discount the future, this reflects a concave utility of life duration function (e.g. a power function with power smaller than 1). The power estimates of the utility function over income may turn out to be constant across questions if we allow for such a generalisation, indicating our rejection is due to an invalid assumption regarding the utility of life duration. This emphasizes the importance of controlling for both utility functions. In addition, the multiplicative model may be valid with a linear utility of life duration, but with the utility function for income having another parametric shape than one belonging to the power family. Its shape may instead be exponential, reflecting constant absolute risk aversion instead of constant relative risk aversion. However, applying an exponential function is more elaborative as it does not give an analytical solution for the exponent and has to be solved numerically for each respondent. In sum, our findings neither necessarily reject the multiplicative or additive shapes of the utility of health and wealth, nor do they necessarily imply the presence of loss aversion; they only indicate that it is inappropriate to model the responses by a combination of a linear utility of life duration function, a power function of wealth, and the assumption of no loss aversion. Further research is required to test which parametric shape best fits lifetime preferences and whether assuming prospect theory causes an improvement in the descriptive validity of individual behaviour.

The significant difference between *X*_1_, the answer to TTO1, and *X*_3_, the answer to QTO, rejects Hypothesis 2 (i.e. $\lambda_{H}$ = $\lambda_{Q}$), and implies a violation of the QALY model. The sign of the difference implies more loss aversion with respect to life duration than with respect to QoL. This finding is consistent with the tendency of people to refuse trading off life years in classical TTO [68]. However, WTP is only higher for TTO1 than QTO under the additive model; in fact, WTP is lower for TTO1 than for QTO when assuming the multiplicative model. The major reason for these contradictory findings seems to be the large number of respondents with negative WTP: for QTO, negative WTP was possible under the multiplicative model, but not under the additive model, resulting in much lower median WTP estimates under the multiplicative model for this procedure. This highlights the importance of the underlying lifetime utility function.

This research clearly has an explorative character. Much work is still needed on the shadow price of a QALY and on the TTO method in general; and clearly also in relation to the potential of the TTO for the income method. Nevertheless, given the existing methodological problems with traditional WTP, alternative approaches should be developed and explored. Furthermore, as described earlier in this discussion, our results open up several new and important areas for future research.

## Appendix A: derivations of predictions additive model

Under the additive utility function (Eq. 1), the case where living $X$ years with the higher income *L* would give equal lifetime utility as the initial scenario with 10 years and income *C*, both in full health (i.e. *Q* = 1), will be evaluated by:

A1 $$D(10) \times Q \times {\text{WTP}}\left( {\text{YFH}} \right) + D(10) \times C = D(X) \times Q \times {\text{WTP}}({\text{YFH}}) + D(X) \times L.$$

Solving Eq. A1 for WTP(YFH) yields:

A2 $${\text{WTP}}\left( {\text{YFH}} \right) = \frac{{D\left( X \right) \times L - D\left( {10} \right) \times C}}{{\left[ {D\left( {10} \right) - D\left( X \right)} \right] \times Q}}.$$

In TTO1, reference-dependence gives the following evaluation:

A3 $$D(10) \times Q \times {\text{WTP}}({\text{YFH}}) + D(10) \times C = \left[ {D\left( {10} \right) + \lambda_{H} \left( {D(X_{1} ) - D(10)} \right)} \right] \times Q \times {\text{WTP}}({\text{YFH}}) + D(X_{1} ) \times L.$$

Solving for *X*_1_ gives:

A4 $$X_{1} = D^{ - 1} \left( {\frac{{D(10) \times \left[ {\lambda_{H} \times Q \times {\text{WTP}}\left( {\text{YFH}} \right) + C} \right]}}{{\lambda_{H} \times Q \times {\text{WTP}}\left( {\text{YFH}} \right) + L}}} \right),$$

which is again increasing in $\lambda_{H}$. Because WTP is increasing in *X*_1_, we predict an overestimation of WTP for a QALY in case of loss aversion.

In TTO2, we obtain the expression below for *X*_2_:

A5 $$X_{2} = D^{ - 1} \left( {\frac{{D(10) \times \lambda_{M} \times (S - C)}}{{\lambda_{H} \times Q \times {\text{WTP}}\left( {\text{YFH}} \right) + C}} + 1} \right).$$

This function is increasing in $\lambda_{H}$ and decreasing in $\lambda_{M}$, yielding the same predictions as for the multiplicative model.

Finally, for QTO, reference-dependence gives:

A6 $$D(10) \times 1 \times {\text{WTP}}({\text{YFH}}) + D(10) \times C = D(10) \times \left[ {1 + \lambda_{Q} \left( {X_{3} - 1} \right)} \right] \times {\text{WTP}}({\text{YFH}}) + D(10) \times L.$$

Solving Eq. A6 for *X*_3_ gives:

A7 $$X_{3} = 1 - \frac{L - C}{{\left( {{\text{WTP}}\left( {\text{YFH}} \right)} \right)\lambda_{Q} }},$$

which is again increasing in $\lambda_{Q} .$

Table 4 presents the WTP estimates under the assumption of the additive model. The observation of Table D2 of more life years given up to move from a subsistence income to their current income, than to move from their current income to a luxury income, clearly translates into a lower WTP estimate in the former task than the latter. In addition, the substantial number of over-traders (resulting in a negative WTP) explains the low median WTP.

**Table 4:** WTP estimates (in €, 2013) additive model

	WTP1 (L–C) No discounting	WTP1 (L–C) 3% discounting	WTP2 (C–S) No discounting	WTP2 (C–S) 3% discounting	WTP3 (L–C QoL)
Disaggregated approach					
Mean	234,465	278,310	55,641	67,669	132,322
Median	20,563	26,971	3542	5730	42,000
Mean (trimmed data)^a^	78,629	96,638	13,381	18,206	138,878
Aggregated approach					
Mean	116,216	140,470	15,236	20,811	97,820
Median	401,250	470,965	5000	8521	62,069
Mean (trimmed data)^a^	86,517	99,153	17,834	21,737	71,493

^a^5% upper and lower values

Regarding the additive model, the mean number of traded life years and the WTP estimates in TTO2 are comparable to those reported by Tilling et al. [29] (their TTO1), while our WTP estimates are higher in TTO1 (their TTO2).^8^ However, it is important to note that the design of the studies differed in two aspects. One difference is that the higher and lower income values in this study were elicited from respondents, whereas these values were given by the experimenters in Tilling et al. [29]. A second difference is that we asked respondents to consider living their remaining lifetime in their current health state, while Tilling et al. [29] instructed respondents to assume to spend the remaining lifetime in full health. Although we corrected for the respondents’ own health by taking their VAS score into account, this may nevertheless have caused differences. Moreover, Tilling et al. used a direct matching procedure, whereas we employed a combination of bisection and matching. However, these differences hold for both versions, so it is not evident why we only observe higher WTP values for the gain version.

## Appendix B: mathematical derivations

Estimation of *α* in TTO1 in case of loss aversion:

$$D(10) \times Q \times C^{\alpha } = \left[ {D(10) + \lambda_{H} (D(X_{1} ) - D(10))} \right] \times Q \times L^{\alpha } \Leftrightarrow \left( {\frac{L}{C}} \right)^{\alpha } = \frac{{D\left( {10} \right)}}{{D\left( {10} \right) + \lambda_{H} \left( {D\left( {X_{1} } \right) - D\left( {10} \right)} \right)}} \Leftrightarrow \alpha \left( {\ln \left( L \right) - \ln \left( C \right)} \right) = \ln \left( {D\left( {10} \right)} \right) - \ln \left( {D\left( {10} \right) + \lambda_{H} \left( {D\left( {X_{1} } \right) - D\left( {10} \right)} \right)} \right) \Leftrightarrow \alpha = \frac{{\ln \left( {D\left( {10} \right)} \right) - \ln \left( {D\left( {10} \right) + \lambda_{H} \left( {D\left( {X_{1} } \right) - D\left( {10} \right)} \right)} \right)}}{\ln \left( L \right) - \ln (C)}.$$

Estimation of α in TTO2 in case of loss aversion:

$$D(10) \times Q \times \left[ {C + \lambda_{M} (S - C)} \right]^{\alpha } = \left[ {D(10) + \lambda_{H} \left( {D(X_{2} ) - D(10)} \right)} \right] \times Q \times C^{\alpha } \Leftrightarrow \left( {\frac{C}{{C + \lambda_{M} \left( {S - C} \right)}}} \right)^{\alpha } = \frac{{D\left( {10} \right)}}{{D\left( {10} \right) + \lambda_{H} \left( {D\left( {X_{2} } \right) - D\left( {10} \right)} \right)}} \Leftrightarrow \alpha \left( {\ln \left( C \right) - \ln \left( {C + \lambda_{M} \left( {S - C} \right)} \right)} \right) = \ln \left( {D\left( {10} \right)} \right) - \ln \left( {D\left( {10} \right) + \lambda_{H} \left( {D\left( {X_{2} } \right) - D\left( {10} \right)} \right)} \right) \Leftrightarrow \alpha = \frac{{\ln \left( {D\left( {10} \right)} \right) - \ln \left( {D\left( {10} \right) + \lambda_{H} \left( {D\left( {X_{2} } \right) - D\left( {10} \right)} \right)} \right)}}{{\ln \left( C \right) - \ln \left( {C + \lambda_{M} \left( {S - C} \right)} \right)}}.$$

Estimation of α in QTO in case of loss aversion

$$D\left( {10} \right) \times 1 \times C^{\alpha } = D\left( {10} \right) \times 1 \times \left[ {1 + \lambda_{Q} \left( {X_{3} - 1} \right)} \right] \times L^{\alpha } \;\;\left( {\frac{L}{C}} \right)^{\alpha } = 1 + \lambda_{Q} \left( {X_{3} - 1} \right)^{ - 1} \Leftrightarrow \alpha = \frac{{ - \ln \left( {1 + \lambda_{Q} \left( {X_{3} - 1} \right)} \right)}}{\ln \left( L \right) - \ln \left( C \right)}.$$

## Appendix C: analysis using disaggregated approach

See Tables 5 and 6.

**Table 5:** Overview

		WTP1 (L–C)	WTP2 (C–S)	WTP3 (L–C QoL)
A	Non-traders^a^	247 (44.9%)	180 (32.7%)	148 (26.9%)
B	Over-traders; negative WTP	77 (14%)	111 (20.2%)	0 (add); 151 (27.5%, mul)
C	Over-traders; trading off all years/quality^a^	1 (0.2%)	8 (1.5%)	2 (0.4%)
D	Zero WTP	12 (2.2%)	59 (10.7%)	0
E	*S* = 0^a^	0	2 (0.4%)	0
F	Net sample size disaggregated approach (550-A-C-E)	302 (54.9%)	360 (65.5%)	400 (72.7%)

*Add* additive model, *mul* multiplicative model

^a^Excluded from analysis

**Table 6:** WTP estimates (in €, 2013) multiplicative model, disaggregated approach

	*α*_1_ no discounting	*α*_1_ 3% discounting	WTP1 (L–C) no discounting	WTP 1 3% discounting	*α*_2_ no discounting	*α*_2_ 3% discounting	WTP2 (C–S) no discounting	WTP 2 3% discounting	*α*_3_	WTP3 (L–C QoL)
Mean	1.01	0.91	7.82e14	8.33e14	1.12	1.02	2,931,121	2,669,288	1.25	1,985,200
Median	0.62	0.55	14,969	15,053	0.86	0.78	2604	2944	0.79	4060
Mean (trimmed data)^a^	0.83	0.75	87,328	78,947	0.94	0.85	11,667	11,655	1.06	28,675

^a^5% upper and lower values removed

## Appendix D: descriptive statistics

See Tables 7 and 8.

**Table 7:** Demographic composition of the sample

Variable	Percentage
Gender (%male)	49.3
Children (%yes)	57.5
Income groups	
<1000 €	14.0
1000 to <2000 €	37.1
2000 to <3000 €	28.9
3000 to <4000 €	13.3
>3999 €	6.7
Education	
Lower	28.6
Middle	41.6
Higher	29.8

**Table 8:** Summary statistics

Variable	Mean	SD	Minimum	Maximum
Age	45.6	15.02	18	75
Health status				
EQ-5D (Dutch tariff)	0.82	0.21	−0.329	1
VAS	76.75	17.75	9	100
Completion time (mins)	16.1	6.2	5	44.9
X_1_	8.03	2.48	0	10
X_2_	7.08	2.83	0	10
X_3_	7.11	2.65	0	10

## Footnotes

1 Eeckhoudt et al. [37] also used an additive function, although they took a linear function over health and a concave function over wealth.

2 Tengstam [39] instead found evidence that marginal utility of income decreases with health.

3 Discounting influences the results, even when health and income are discounted at the same rate, because years given up necessarily occur at the end of the timeframe, whereas one can immediately start enjoying the higher income. Hence, years given up are more heavily influenced by discounting.

4 Incomes were expressed in monthly terms in the questionnaire, because most people are used getting their income once a month. However, for the analysis we transformed monthly incomes into yearly incomes, since we estimate willingness to pay for healthy life years. Hence, we refer to *C*, *L* and *S* as yearly amounts throughout this paper.

5 The exponential model performed worse than the power model and is not presented in the paper. The results of this model are available from the authors upon request. For loss aversion, we implemented a loss aversion coefficient of 1.18 for both health and money, which was the median loss aversion index for life years reported by Attema et al. [45].

6 For the remaining 34 respondents, a ratio could not be computed because their denominator (L + S-2C) equalled 0.

7 This may also occur in a non-exponential fashion, such as according to a hyperbolic discounting function.
